# Serum Fourier-Transform Infrared Spectroscopy with Machine Learning for Screening of Pediatric Acute Lymphoblastic Leukemia: A Proof-of-Concept Study

**DOI:** 10.3390/cancers17213548

**Published:** 2025-11-01

**Authors:** Aneta Kowal, Paweł Jakubczyk, Wioletta Bal, Zuzanna Piasecka, Klaudia Szuler, Kornelia Łach, Katarzyna Sopel, Józef Cebulski, Radosław Chaber

**Affiliations:** 1 Doctoral School, Institute of Medical Science, University of Rzeszów, 35-959 Rzeszów, Poland; anet.kow@wp.pl; 2 Institute of Physics, Faculty of Exact and Technical Sciences, University of Rzeszow, 35-310 Rzeszów, Poland; pjakubczyk@ur.edu.pl (P.J.); jcebulski@ur.edu.pl (J.C.); 3 Department of Pediatrics, Faculty of Medicine, University of Rzeszow, 35-310 Rzeszow, Poland; wbal@ur.edu.pl (W.B.); zuz.pia@wp.pl (Z.P.); kszuler@ur.edu.pl (K.S.); 4 Clinic of Pediatric Oncology and Hematology, Provincial Clinical Hospital 2, 35-301 Rzeszów, Poland; 5 Department of Medical Chemistry and Matabolomics, Faculty of Medicine, University of Rzeszów, 35-959 Rzeszów, Poland; kolach@ur.edu.pl; 6 Laboratory Diagnostics Department., Independent Public Healthcare Facility in Przeworsk, 37-200 Przeworsk, Poland; ksopel@spzoz-przeworsk.pl; 7 Department of Medicine, Faculty of Medicine and Health Sciences, University of Applied Sciences in Tarnow, 33-100 Tarnów, Poland

**Keywords:** Fourier-transform infrared spectroscopy, acute lymphoblastic leukemia, pediatric oncology, serum biomarkers, vibrational spectroscopy, diagnostics

## Abstract

**Simple Summary:**

Acute lymphoblastic leukemia is the most common pediatric malignancy and its diagnosis still relies on bone-marrow evaluation, an invasive and technically demanding procedure. There is ongoing interest in developing minimally invasive methods that could support earlier recognition of the disease. In this study, we assessed whether an infrared spectroscopy of blood serum can detect biochemical differences between children with leukemia and those without. Characteristic spectral shifts were observed in regions associated with proteins, lipids, and nucleic acids. Based on these patterns, machine-learning models achieved moderate accuracy in distinguishing leukemia from controls. Although the method is fast, cost-effective, and uses minimal serum material, the present results remain preliminary. Validation in larger, multi-center cohorts will be essential to establish reliability and clinical value before potential adoption in pediatric diagnostics.

**Abstract:**

Background: Acute lymphoblastic leukemia (ALL) is the most common childhood malignancy, yet diagnosis still relies primarily on invasive bone-marrow procedures and advanced laboratory assays. Non-invasive, rapid, and cost-effective tools remain an unmet need. Fourier-transform infrared (FTIR) spectroscopy has shown promise for detecting cancer-associated biochemical changes in biofluids and cells. Methods: Serum from pediatric ALL patients and controls (*n* = 103; ALL = 45, controls = 58: healthy = 14, hematology controls = 44 with anemia, thrombocytopenia, leukopenia, and pancytopenia) was analyzed using FTIR. Spectra (800–1800, 2800–3500 cm^−1^) were preprocessed with baseline correction, derivative filtering, and normalization. Group differences were assessed statistically, and logistic regression with stratified 10-fold cross-validation was applied; Receiver operating characteristic (ROC)\precision–recall (PR) analyses were based on out-of-fold predictions. Results: Distinct spectral alterations were observed between ALL and controls. Leukemia samples showed higher amide I (~1640 cm^−1^) and amide II (~1545 cm^−1^) absorbance, lower lipid-related bands (~1450, ~2920 cm^−1^), and increased nucleic-acid–associated signals (~1080 cm^−1^). Differences were significant (*q* < 0.05) with moderate effect sizes. Logistic regression achieved area under the curve (AUC) ≈ 0.80 with sensitivity ~0.73–0.84 across practical decision thresholds (0.50 → 0.30) and higher recall attainable at the expense of specificity. Principal component analysis (PCA)\hierarchical cluster analysis (HCA) indicated partial but consistent group separation, aligning with supervised performance. Conclusions: Serum FTIR spectroscopy shows promise for distinguishing pediatric ALL from controls by reflecting disease-related metabolic changes. The technique is rapid, label-free, and requires only small serum volumes. Our findings represent proof-of-concept, and validation in larger, multi-center studies is needed before clinical implementation can be considered.

## 1. Introduction

Acute lymphoblastic leukemia (ALL) is the most common pediatric malignancy, accounting for approximately 25–30% of cancers in children under 15 years of age [1]. It arises from malignant transformation of lymphoid progenitors, most frequently of B-lineage, and is characterized by clonal expansion of immature lymphoblasts in bone marrow and peripheral blood.

The clinical presentation of ALL is heterogeneous. Some patients present with mild cytopenias affecting a single lineage, whereas others present with profound cytopenias or even extreme leukocytosis. In particular, hyperleukocytosis at diagnosis can result in life-threatening complications such as leukostasis, tumor lysis syndrome, and disseminated intravascular coagulation, making rapid diagnosis and treatment initiation essential [2]. Differential diagnosis is broad and, in pediatrics, must include among others severe aplastic anemia (SAA), myelodysplastic syndromes (MDS), and post-infectious bone-marrow suppression, but sometimes it is only an isolated cytopenia—anemia, neutropenia, or thrombocytopenia [3].

The diagnostic gold standard for ALL remains bone-marrow biopsy with cytological and immunophenotypic evaluation [4]. These methods combined with genetic profiling, achieve near-complete diagnostic accuracy (sensitivity and specificity approaching 100% when performed by experienced hematopathologists). However, these methods have important limitations: (1) Invasiveness: bone-marrow procedures are painful, require sedation or anesthesia in children, and carry risk of complications such as bleeding or infection; (2) resource intensity: expert interpretation, specialized laboratory facilities, and turnaround time of several days are required; (3) limited applicability for early screening: bone-marrow biopsy is not suitable for evaluating children with mild or nonspecific cytopenias where leukemia is only one of many differential diagnoses. These limitations create a clinical need for minimally invasive, rapid screening tools that can identify high-risk patients who warrant definitive bone-marrow examination.

However, clinical and laboratory findings are not always sufficient to immediately justify such an invasive procedure, particularly in children with subtle or non-specific blood count abnormalities. This diagnostic uncertainty may delay definitive testing and therapeutic intervention. Therefore, there is a strong rationale for developing a simple, low-cost screening assay that could be integrated into routine clinical workflows, ideally performed in parallel with standard complete blood counts. Such an approach could help identify children at high risk for ALL earlier, prompting timely referral for bone-marrow evaluation.

Vibrational spectroscopies, including Fourier-transform infrared (FT-IR) and Raman spectroscopy, probe the vibrational modes of biomolecules, generating unique “fingerprints” of biological samples [5,6]. These methods are rapid, label-free, non-destructive, and require only microliter amounts of biofluids. The resulting spectra reflect the relative abundance of proteins, lipids, carbohydrates, and nucleic acids, enabling sensitive detection of biochemical changes associated with disease states [7].

In oncology, vibrational spectroscopy has been applied successfully to classify biofluids, tissues, and cells, assisting in cancer detection, grading, prognosis, and treatment monitoring [8,9]. Its main advantages are minimal sample preparation, short acquisition times, low consumable costs, and the possibility of integration into routine laboratory workflows.

Given these properties, serum-based FT-IR spectroscopy represents a promising adjunct to current hematology diagnostics. In the present study, we tested whether serum FT-IR spectra can differentiate pediatric ALL from healthy and non-ALL hematology controls, thereby evaluating its potential as a screening tool in pediatric oncology.

## 2. Materials and Methods

### 2.1. Patient Material and Ethical Approval

Between 2016 and 2024, a total of 91 pediatric patients were enrolled at the Department of Pediatric Oncology and Hematology, Clinical Provincial Hospital No. 2, Rzeszów, Poland. Of these, 45 were diagnosed with ALL and 44 presented with non-ALL hematological disorders, including iron-deficiency anemia (*n* = 6), leukopenia (*n* = 16), pancytopenia (*n* = 8), and thrombocytopenia (*n* = 13). The control group consisted of 14 healthy children recruited at the Department of Laboratory Diagnostics, Przeworsk, Poland. Inclusion criteria for healthy controls were: age under 18 years, absence of acute infection within the preceding two weeks, no history of chronic disease or long-term pharmacotherapy, and no prior malignancy. Patient characteristics are summarized in Table 1.

Blood samples from the control group were obtained during other routine diagnostic procedures, and all children had normal peripheral blood morphology. In patients with ALL, peripheral blood and bone-marrow aspirates were collected as part of standard hematological diagnostics to confirm the diagnosis. In children with non-ALL hematological disorders, blood samples were collected as part of their routine diagnostic work-up. The study was approved by the Bioethics Committee of the University of Rzeszów (Resolution No. 1/01/2020 of 30 January 2020 and No. 2022/045/W of 4 May 2022).

The diagnosis of ALL was established based on cytological examination and immunophenotyping, which identified 43 cases of B-lineage ALL and 5 cases of T-lineage ALL. The mean bone-marrow blast percentage was 79% (range 20–96%, median 85%), with only 9 patients presenting with values below 50%. Diagnosis and treatment were conducted according to established international protocols, including ALL IC-BFM 2009 (*n* = 14), EsPhALL2009 (*n* = 2), AIEOP-BFM ALL 2017 (*n* = 22), and AIEOP-BFM ALL 2017 Poland (*n* = 10). Nine patients were classified as high-risk, three of whom carried unfavorable prognostic markers (BCR-ABL fusion, *n* = 2; KMT2A rearrangement, *n* = 1). Four patients with high minimal residual disease (PCR-MRD) TP1 > 5 × 10^−4^ were categorized as early high-risk. Three patients failed to achieve remission after 33 days, and two showed elevated PCR-MRD TP2 > 5 × 10^−4^ after consolidation. Relapse occurred in three cases (one high-risk, two non–high-risk), and one death was recorded (relapse, non–high-risk).

### 2.2. Sample Preparation and Spectral Acquisition

Peripheral blood was collected into coagulation-activated syringes (S-Monovette, Sarstedt, Nümbrecht, Germany). Serum was separated by centrifugation at 3000 g for 10 min, followed by 5000 rpm for 5 min. Samples were processed within 2 h and stored at −80 °C until analysis.

All serum samples were subjected to a single freeze–thaw cycle prior to FTIR analysis. Samples were measured in randomized order to minimize potential batch effects.

For FT-IR measurements, 20 µL aliquots of serum were deposited onto polished CaF_2_ windows (Crystan, Poole, UK) and dried in a dust-free desiccator for 3 h before measurement. Spectra were acquired in attenuated total reflection (ATR) mode using a Bruker Vertex 70v spectrometer (Bruker, Katowice, Poland) equipped with an MCT detector and single-reflection diamond ATR accessory.

For diagnostic and chemometric analyses, spectra were evaluated within the 800–1800 cm^−1^ and 2800–3500 cm^−1^ regions, encompassing major biochemical bands in serum.

For the repeatability and stability study, a slightly narrower window (900–1800 cm^−1^ and 2800–3050 cm^−1^) was applied to reduce the influence of water-vapor interference and high-frequency noise above 3050 cm^−1^.

This refinement improves the sensitivity of stability-related metrics while preserving all diagnostically relevant bands (amide I/II and CH_2_ stretching).

The same preprocessing steps were used for both spectral masks to ensure methodological consistency.

Acquisition parameters:

- range: 4000–400 cm^−1^

- resolution: 4 cm^−1^

- scans: 64 per sample and background

- mode: attenuated total reflection (ATR)

- atmosphere: automatic compensation for CO_2_ (2400–2300 cm^−1^) and H_2_O (2100–1250 cm^−1^).

Each spectrum was recorded in triplicate. Raw spectra were preprocessed in OPUS (Bruker) for vector normalization and rubberband baseline correction (64 points).

### 2.3. Computational Preprocessing and Analysis

Subsequent preprocessing and classification were conducted in Python 3.10. Spectral windows 800–1800 and 2800–3500 cm^−1^ were retained; atmospheric regions were masked. Baseline correction used Asymmetric Least Squares (AsLS, *λ* = 1 × 10^5^, *p* = 0.01, 10 iterations) [10]. Baseline correction employed a two-stage approach: first, an initial rubber-band baseline correction (64 points) to remove large-scale background curvature, followed by Asymmetric Least Squares (AsLS) refinement (*λ* = 1 × 10^5^, *p* = 0.01, 10 iterations) [10].

This methodology was quantitatively validated for feature preservation, confirming: peak position stability at biomarker regions (amide I: 1640 cm^−1^; amide II: 1545 cm^−1^; lipids: 2920 cm^−1^), maximum absolute difference ≤0.0067 AU at key bands, full-spectrum RMSD of 0.0082 AU versus single-step AsLS, and re-correction stability (maximum deviation ≤0.0008 AU upon reprocessing) (Table S1). These variations represent <1% intensity change—below biological variability thresholds (± 0.05 AU) and clinical significance limits.

Savitzky–Golay second derivative (window = 11, polynomial order = 3) was applied to enhance spectral contrast [11]. Standard Normal Variate (SNV) normalization was subsequently performed [12]. These preprocessing steps were selected based on established protocols in biofluid FTIR spectroscopy and have been validated across numerous serum and plasma studies to ensure reproducibility and comparability with published workflows. Peak analysis extracted amplitude, peak position, and normalized amplitude relative to the Amide I band at 1639 cm^−1^. Group differences were assessed using Welch’s *t*-test with Benjamini–Hochberg false discovery rate correction (*q* < 0.05) [13]. Effect sizes were reported as Cohen’s d.

The primary classifier was logistic regression with L2 regularization and class balancing (class_weight = “balanced”), implemented in scikit-learn [14]. In addition, Support Vector Machine (linear kernel), Random Forest, and Linear Discriminant Analysis were also applied to evaluate the robustness of spectral discrimination. Model performance was evaluated using stratified 10-fold cross-validation to ensure balanced representation of both groups in each fold. Out-of-fold predicted probabilities were used to generate ROC and precision–recall curves as well as confusion matrices. Different thresholds (0.5, 0.4, 0.3, and ~0.47) were compared to assess the recall–specificity trade-off. Bootstrap resampling (*n* = 2000) was applied to estimate 95% confidence intervals for AUC, recall, specificity, precision, accuracy, and F1 score. Performance was assessed using sensitivity, specificity, accuracy, precision, F1 score, ROC AUC, balanced accuracy, and Matthews correlation coefficient (MCC). Metric stability across folds was illustrated using violin and box plots. Dimensionality reduction and clustering were explored with PCA and hierarchical cluster analysis (Ward linkage on the first 10 PCs).

To quantify instrument error and model stability across the three FT-IR replicates per patient, we generated out-of-fold predictions for each replicate using patient-grouped cross-validation (GroupKFold) so that replicates from the same patient were never split across train/test folds. Spectral windows (900–1800 and 2800–3050 cm^−1^), Savitzky–Golay second derivative, and SNV were used as in the main pipeline. The full protocol is provided in Supplementary Methods “Replicate-level prediction variability”.

All analyses were conducted in Python (v3.11) using NumPy (v1.26) [15], pandas (v2.1) [16], SciPy (v1.11) [17], and scikit-learn (v1.3) [14]. Visualizations were prepared with matplotlib [18] and seaborn [19]. Manuscript preparation followed established protocols for FTIR biofluid analysis [5,6,20].

In addition, statistical analyses, computational procedures, and figure generation were supported by generative artificial intelligence (GenAI). All GenAI-assisted results were independently validated and cross-checked using MATLAB Version: 24.2.0.2806996 (R2024b) to ensure accuracy and reproducibility.

## 3. Results

### 3.1. Spectral Preprocessing and Global Overview

**Figure 1:**
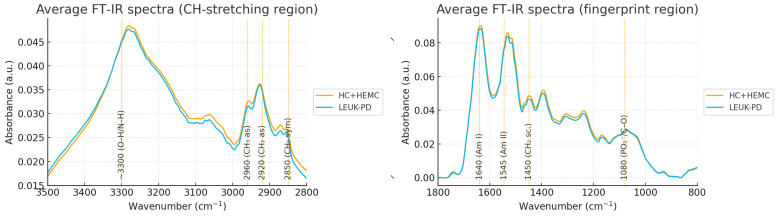
Average vector-normalized FT-IR spectra of serum samples. The blue curve represents the combined healthy control (HC) and hematological control (HEMC) groups, while the red curve corresponds to patients with leukemia at diagnosis (LEUK-PD). The spectral region between 1800 and 2800 cm^−1^ was omitted due to the absence of relevant absorption bands, as indicated by the axis break. The figure provides a qualitative overview of overall spectral similarity between groups; quantitative intensity differences (including lipid band reductions at 1450 and 2920 cm^−1^) were evaluated using statistical peak-level analysis (Table 2).

After excluding atmospheric windows and applying the full preprocessing pipeline (asymmetric least squares baseline correction, Savitzky–Golay second derivative [11], and standard normal variate normalization), a total of 103 serum spectra were retained for analysis. Each sample was measured in triplicate, and replicate spectra showed nearly identical profiles across the full spectral range, confirming excellent reproducibility of the FTIR measurements. Visual inspection of the group-averaged spectra revealed systematic differences between LEUK-PD and CONTROLS (combined HC and HEMC groups), most pronounced in protein- and lipid-associated regions. Specifically, LEUK-PD samples exhibited increased absorption in the amide I (~1640 cm^−1^) and amide II (~1545 cm^−1^) bands, whereas lipid-associated CH_2_ vibrations at 1450 cm^−1^ and 2920 cm^−1^ showed reduced intensity (Figure 1). These broad spectral shifts point to an altered protein-to-lipid balance in leukemic serum, consistent with metabolic reprogramming and enhanced cellular turnover.

**Table 2:** Discriminatory FT-IR peaks (LEUK-PD vs. CONTROLS). Legend: Arrows indicate relative change in LEUK-PD. Peak assignments based on vibrational band libraries [10,11,12,13,14].

Peak (cm^−1^)	Tentative Assignment	Direction (LEUK-PD vs. CONTROLS)	Effect Size (Cohen’s d)	*q*-Value
1640 (amide I)	C=O stretching of proteins	↑ LEUK-PD	0.85	0.004
1545 (amide II)	N–H bending, C–N stretching	↑ LEUK-PD	0.62	0.021
1450 (CH_2_ scissoring)	Lipids/protein backbone	↓ LEUK-PD	0.55	0.030
2920 (CH_2_ stretch)	Lipids	↓ LEUK-PD	0.70	0.015
1080 (C–O, PO_2_^−^)	Nucleic acids/carbohydrates	↑ LEUK-PD	0.58	0.040

### 3.2. Peak-Level Analysis

Fifteen reproducible peaks were identified in the fingerprint region (900–1800 cm^−1^) and CH-stretching region (2800–3050 cm^−1^). Statistical comparison using Welch’s *t*-test with FDR correction revealed five discriminatory bands with *q* < 0.05 (Table 2).

The strongest separation was observed in the amide I band (1640 cm^−1^), which was significantly higher in LEUK-PD (Cohen’s *d* = 0.85). Amide II (1545 cm^−1^) was likewise elevated, suggesting global increases in serum protein content. In contrast, lipid-associated bands (1450 and 2920 cm^−1^) were reduced in LEUK-PD, reflecting alterations in lipid metabolism. An additional discriminatory band was observed at 1080 cm^−1^, linked to nucleic acids and carbohydrates, which was increased in LEUK-PD, consistent with higher nucleic acid turnover in proliferating leukemic blasts. These findings confirm that a small subset of spectral features can capture biologically relevant differences between leukemia and non-leukemic states, reflecting underlying alterations in serum composition.

### 3.3. Multivariate Classification

**Figure 2:**
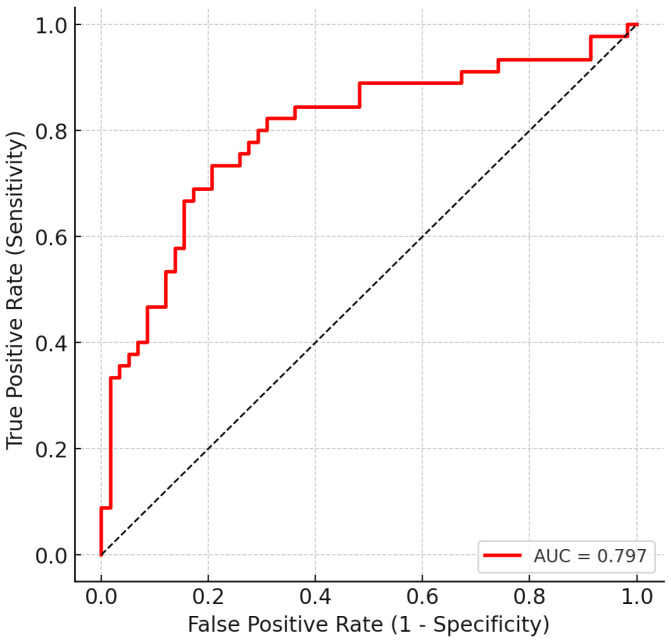
Receiver operating characteristic (ROC) curve for discrimination of LEUK-PD vs. CONTROLS based on full preprocessed FT-IR spectra. Results shown are out-of-fold predictions from 10-fold stratified cross-validation (balanced logistic regression). The classifier achieved an AUC of 0.80. The shaded diagonal represents no-skill performance.

**Figure 3:**
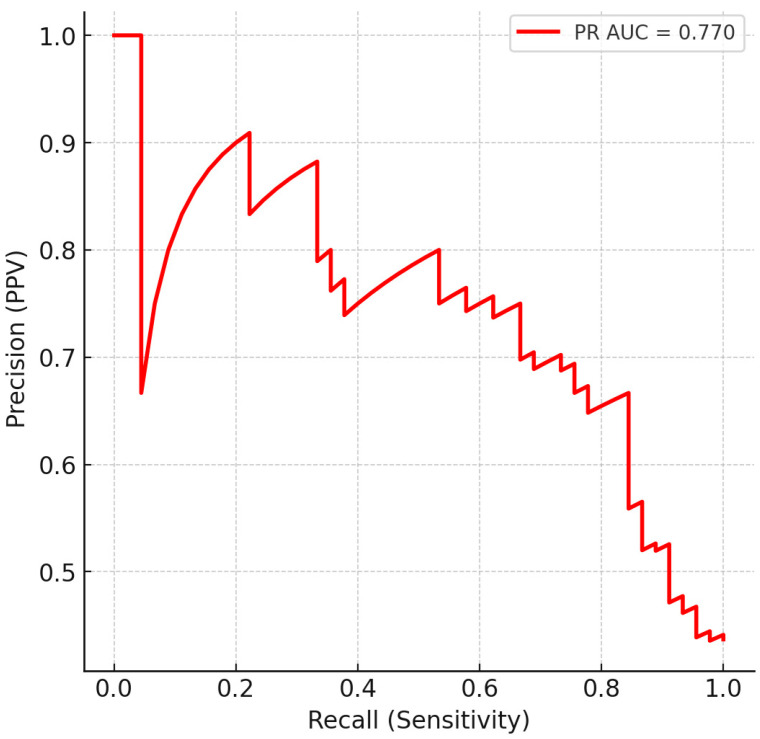
Precision–Recall (PR) curve for classification of LEUK-PD vs. CONTROLS using out-of-fold predictions from 10-fold stratified cross-validation. The model achieved a PR AUC of 0.77. PR analysis illustrates the trade-off between sensitivity (recall) and positive predictive value (precision), relevant for screening-oriented applications.

We next evaluated whether the full preprocessed spectra could discriminate LEUK-PD from CONTROLS (HC + HEMC) using supervised machine learning. Logistic regression with L2 regularization and class weighting was trained in a nested cross-validation framework. The classifier achieved a mean AUC of 0.80 (95% CI: 0.71–0.87) across outer folds (Figure 2). At the conventional decision threshold of 0.5, the model yielded a sensitivity of 0.74 and a specificity of 0.72. Lowering the threshold to 0.4 improved sensitivity to 0.83, at the cost of a reduction in specificity to 0.64. Among the tested operating points, a threshold of 0.47 provided the most balanced configuration (sensitivity 0.78, specificity 0.71, F1 = 0.72), and was therefore selected as the representative screening-oriented setting (Table 3, Figure 3). Precision–recall analysis further confirmed that recall could be tuned up to 0.90, albeit with reduced specificity, underscoring the flexibility of the model for different clinical contexts. From a screening standpoint, such adaptability is crucial, as minimizing false negatives takes priority even at the cost of moderate increases in false positives.

### 3.4. Confusion Matrix and Operating Characteristics

**Figure 4:**
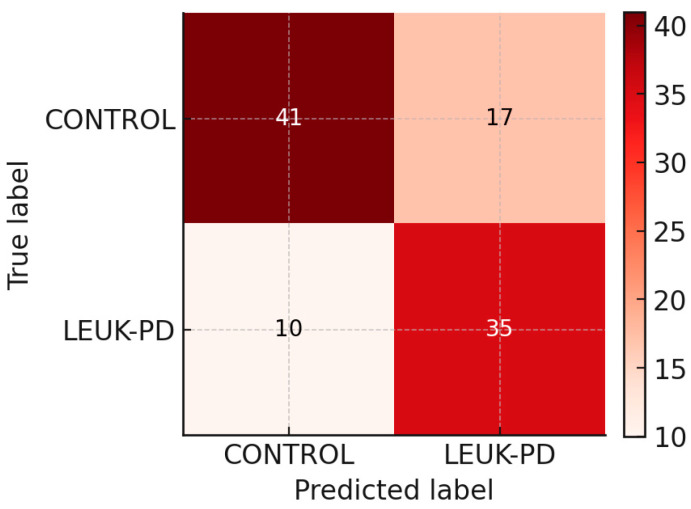
Confusion matrix at probability threshold 0.47 for classification of LEUK-PD vs. CONTROLS based on preprocessed FT-IR spectra. Results are derived from out-of-fold predictions obtained in 10-fold stratified cross-validation (balanced logistic regression). At this operating point, the classifier achieved 78% sensitivity (35/45 LEUK-PD correctly identified) and 71% specificity (41/58 controls correctly classified), illustrating a balanced trade-off suitable for screening-oriented applications.

The confusion matrix at threshold 0.47 (Figure 4) illustrates the balance between true-positive detection of leukemia and false-positive classification of controls. At this operating point, the classifier achieved 78% sensitivity (35/45 LEUK-PD correctly identified) and 71% specificity (41/58 controls correctly classified). This configuration provides a practical compromise for screening applications, ensuring that most leukemic cases are detected while maintaining an acceptable false-positive rate.

### 3.5. PCA and HCA

**Figure 5:**
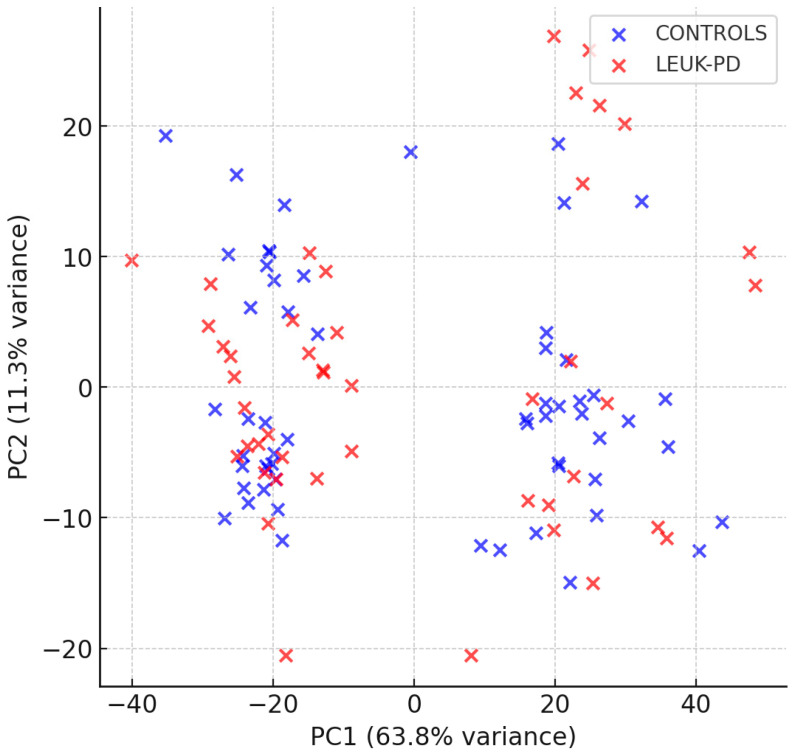
Principal component analysis (PCA) of preprocessed FT-IR spectra. The first two components explained ~75% of the total variance (PC1 = 63.8%, PC2 = 11.3%). LEUK-PD samples (red) tended to shift relative to CONTROLS (blue) along PC1, consistent with biochemical differences in protein- and lipid-associated regions.

Unsupervised group structure was first explored using principal component analysis (PCA). The first two principal components explained ~75% of the total variance (PC1 = 63.8%, PC2 = 11.3%). LEUK-PD samples tended to shift relative to CONTROLS along PC1, consistent with dominant contributions from protein (amide I/II) and lipid-associated bands (Figure 5). Although the univariate comparison of PC1 scores alone was not significant (Welch’s *t*-test *p* = 0.82; Figure S1), the multivariate pattern captured by PCA—supported by loading profiles pointing to amide and lipid regions (Figure S2)—indicates a coherent biochemical shift between groups.

**Figure 6:**
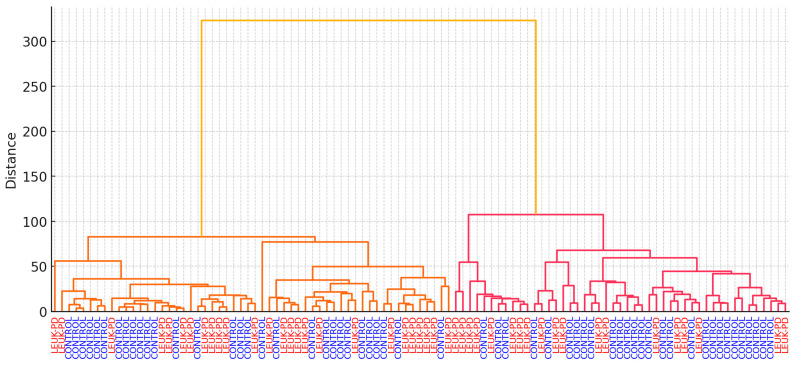
Hierarchical cluster analysis (HCA) using Ward’s linkage on the first 10 principal components. Two major branches enriched for LEUK-PD (red) and CONTROLS (blue) were observed, with some intermixing. Colored branches and labels highlight the unsupervised separation of disease and control spectra.

Hierarchical cluster analysis (HCA) using Ward’s linkage on the first 10 PCs further corroborated these findings, yielding two major branches enriched for LEUK-PD with controls largely segregating into separate clusters (Figure 6). Despite some intermixing, the clustering pattern and the PC-score heatmap (Figure S3) underscore that serum FT-IR spectra encode clinically relevant biochemical signatures associated with ALL. From a clinical perspective, such unsupervised separation complements the supervised model, suggesting that disease-related alterations in the protein–lipid balance are sufficiently pronounced to emerge without class labels, thereby supporting feasibility for screening-oriented applications.

### 3.6. Stability Analysis

**Figure 7:**
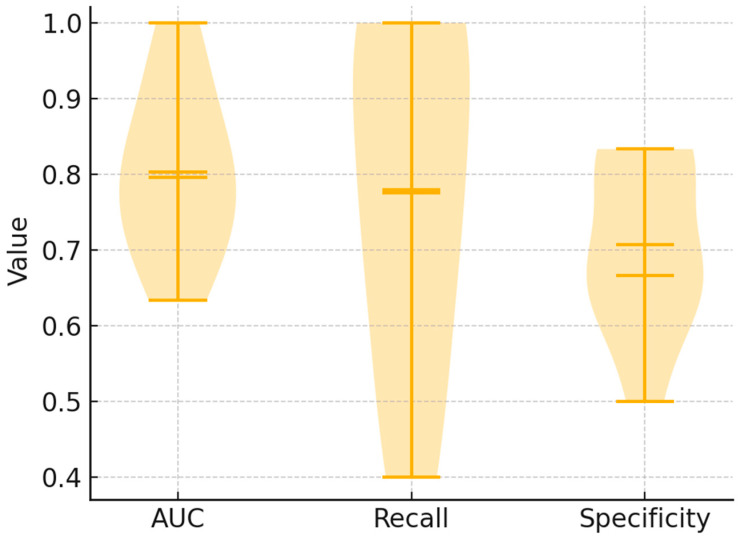
Per-fold distributions of classification performance in 10-fold stratified cross-validation. Violin plots depict the distribution of AUC, recall, and specificity values at the screening-oriented threshold of 0.47. Boxes indicate the interquartile range, while the central line denotes the median.

**Figure 8:**
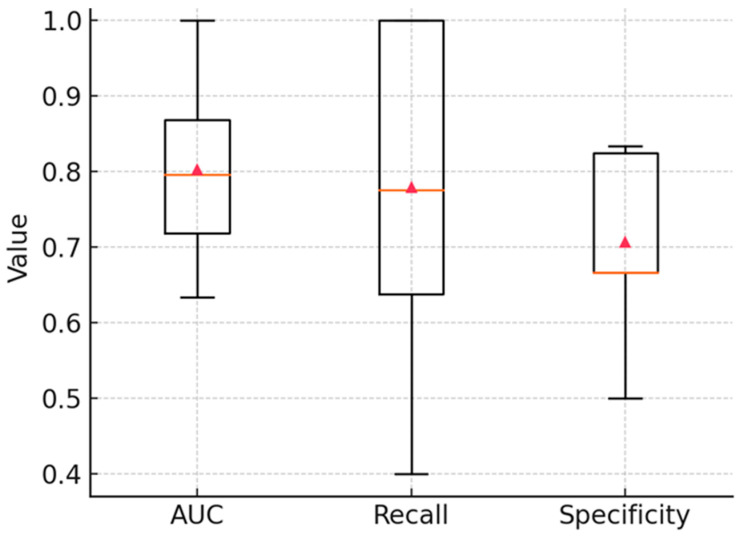
Per-fold distributions of classification performance in 10-fold stratified cross-validation. Box plots summarize AUC, recall, and specificity values at the threshold of 0.47. Red triangles mark the mean across folds. Results demonstrate stable classifier performance with median AUC ≈ 0.83, recall ≈ 0.78, and specificity ≈ 0.67.

Finally, to assess model robustness under the screening-oriented operating point, we evaluated per-fold performance distributions in 10-fold stratified cross-validation after the full preprocessing pipeline (AsLS baseline correction → Savitzky–Golay second derivative → SNV; spectral mask 900–1800 and 2800–3050 cm^−1^). Violin and box plots of AUC, recall, and specificity at the fixed threshold of 0.47 (Figure 7 and Figure 8) showed consistent behavior across outer folds, with mean AUC of 0.81 (median 0.83), mean recall of 0.78 (median 0.78), and mean specificity of 0.71 (median 0.67). These results confirm reproducible classification performance at the selected screening threshold. From a statistical perspective, the narrow interquartile ranges demonstrate model stability across resampled partitions, indicating that the discrimination is not attributable to chance. Clinically, the observed balance between high recall (minimizing false negatives) and acceptable specificity (limiting false positives) underscores the potential of FT-IR spectroscopy as a reliable screening tool for early leukemia detection in pediatric cohorts.

Across 103 patients (308 replicates), replicate-level predictions were highly consistent. Inter-replicate agreement was excellent (probabilities: ICC (2, 1) = 0.870; 95% CI 0.800–0.919; logits: ICC (2, 1) = 0.872; 95% CI 0.805–0.916; *n* = 96 complete triplicates). Within-patient variability was modest (probabilities: median RSD = 6.03%, IQR 2.59–14.05%; logits: median SD = 0.198, IQR 0.064–0.359). Bland–Altman analyses showed negligible bias and ~±0.20 95% limits of agreement for probabilities. Full statistics and figures are provided in Supplementary Figures S4–S11 and Supplementary Table S1.

### 3.7. Alternative Classification Methods for Spectral Discrimination

To verify whether the discriminative performance observed with logistic regression was dependent on the choice of algorithm, we additionally evaluated three commonly used classifiers: Support Vector Machine (SVM, linear kernel), Random Forest (RF), and Linear Discriminant Analysis (LDA). All models were trained on fully preprocessed FTIR spectra (AsLS baseline correction, Savitzky–Golay second derivative, standard normal variate normalization) using stratified 10-fold cross-validation.

All classifiers achieved consistent performance with AUC values in the 0.74–0.81 range (Table 4). LDA performed best in terms of balanced accuracy (0.78) and MCC (0.56), indicating a strong equilibrium between sensitivity and specificity. Random Forest also achieved AUC ~0.81, though with slightly lower sensitivity. In contrast, SVM with a linear kernel yielded somewhat lower performance (AUC = 0.74).

**Figure 9:**
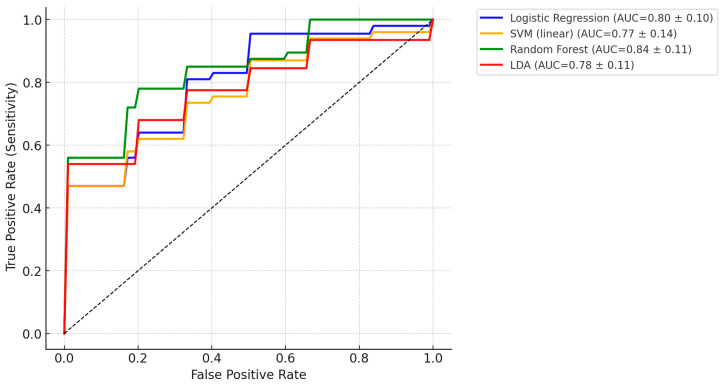
Average receiver operating characteristic (ROC) curves obtained from stratified 10-fold cross-validation for four classifiers: Logistic Regression, Support Vector Machine (linear kernel), Random Forest, and Linear Discriminant Analysis. Each curve represents the mean performance across folds, with area under the curve (AUC) values indicated in the legend (mean ± standard deviation). Logistic Regression and Random Forest achieved the highest AUC values (~0.80–0.84), while LDA performed comparably (AUC ~0.78). SVM with a linear kernel showed slightly lower performance (AUC ~0.77). These results demonstrate that FTIR-based spectral discrimination between LEUK-PD and controls is robust across different classification algorithms.

Figure 9 presents the average ROC curves of the three alternative classifiers, demonstrating that the classification performance was robust and reproducible regardless of algorithm choice. The convergence of results across methods supports the interpretation that the discriminative signal originates from reproducible biochemical differences rather than model-specific artifacts.

## 4. Discussion

This study demonstrates that serum-based FT-IR spectroscopy can discriminate ALL from both healthy and non-leukemic hematology controls in approximately 80% of cases, highlighting its potential as a non-invasive adjunct for early disease screening. The observed spectral shifts, particularly the elevation of protein-associated amide I (~1640 cm^−1^) and amide II (~1545 cm^−1^) bands alongside the reduction in lipid-related CH_2_ vibrations (~1450 and 2920 cm^−1^), are consistent with metabolic reprogramming characteristic of malignant proliferation. Increased intensity at ~1080 cm^−1^, attributed to nucleic acids and carbohydrates, further reflects heightened nucleic acid turnover associated with rapid leukemic blast proliferation. Together, these findings indicate that a relatively small set of spectral features captures biologically meaningful differences between leukemic and non-leukemic serum profiles.

The discriminatory power of these biochemical signatures was supported by supervised classification. Balanced logistic regression achieved a mean AUC of 0.80 (95% CI: 0.71–0.87) across outer folds; at the representative threshold of 0.47, performance was sensitivity 0.78, specificity 0.71, F1 = 0.72, with alternative thresholds (0.50/0.40/0.30) tracing the expected sensitivity–specificity trade-offs. Precision–recall analysis yielded PR AUC ≈ 0.77 and confirmed that recall can be tuned up to ~0.90 at the expense of specificity—an option relevant for screening-oriented operation where minimizing false negatives is prioritized.

Complementary unsupervised analyses, including PCA and HCA, showed that leukemia cases tend to form partially distinct clusters with some intermixing, reinforcing the robustness of the biochemical divergence observed in the spectra. This is concordant with PCA explaining ~75% of variance (PC1 ≈ 63.8%, PC2 ≈ 11.3%) and HCA producing two main branches enriched for LEUK-PD while retaining some intermixing, consistent with the moderate—but reproducible—group separation observed in supervised results.

Finally, alternative classifiers (linear SVM, Random Forest, LDA) corroborated robustness: performance clustered in the AUC ≈ 0.74–0.81 range; LDA provided the highest balanced accuracy and MCC, RF matched AUC ~0.81 with slightly lower sensitivity, and linear SVM trailed modestly. Convergence across algorithms indicates that discrimination arises from reproducible biochemical differences rather than model-specific artifacts.

The relevance of FTIR spectroscopy as a pediatric leukemia screening tool has also been suggested by our previous preliminary, serum-based study, which reported significant differences in protein secondary structure (lower %β-sheet + β-turn in ALL) and lipid-associated features (reduced 2965/1645 cm^−1^ ratio) between ALL patients and controls, with an AdaBoost predictive model achieving 85% accuracy in leave-one-out cross-validation (Chaber et al., 2021 [21]). Taken together with our cohort-based analysis, these data indicate consistent FTIR-derived trends (protein/lipid balance, amide I region) across independent patient sets, while underscoring the need for validation in larger cohorts.

Building on this, our findings are in line with a growing body of evidence demonstrating the potential of infrared spectroscopy for hematological diagnostics. Several reviews and authoritative overviews have emphasized the non-invasive, rapid, and cost-effective nature of FTIR, highlighting its capacity to capture subtle biochemical alterations in blood cells and biofluids that accompany malignancy (Delrue et al. [22]; Liu et al. [23]; Mostaço-Guidolin & Bachmann [24]). Importantly, the discriminative features we identified—particularly shifts in protein-to-lipid balance and nucleic-acid–associated bands—are consistent with alterations reported across experimental and clinical leukemia studies, including documented changes in protein secondary structure and DNA-related signals [23,24].

For instance, AI-enhanced ATR-FTIR applied to fixed, unstained peripheral blood smears recently achieved diagnostic accuracies above 80% (e.g., SVM accuracy ~83%, AUC ~90.8%), with peak-level differences most prominent in amide I/II, glycogen, and phosphorylated-protein regions (Lee et al. [25]). Notably, in that peripheral blood smears (PBS) setting leukemia samples showed higher glycogen (~1032–1073 cm^−1^) and phosphorylated-protein (~892–894 cm^−1^) absorbance but lower amide I/II and nucleic-acid–related bands than controls—directionality explicitly described in the results and assignments [25]. Likewise, long-term reviews of FTIR in leukemia consistently report biochemical markers distinguishing leukemic from normal cells, particularly DNA/PO_2_^−^-associated bands (~1000–1150 cm^−1^) and protein-conformation changes in the amide region (amide I/II), underscoring FTIR’s sensitivity to nucleic acids and protein secondary structure [22,24].

These observations parallel our results in serum, where increased absorption near ~1640 cm^−1^ (amide I) and ~1080 cm^−1^ (nucleic acids/carbohydrates) co-occurred with reduced lipid-associated bands (~1450, ~2920 cm^−1^), indicating a protein- and nucleic-acid–shifted profile relative to controls. They are complementary to the PBS ATR-FTIR findings of Lee et al., in which leukemia samples exhibited higher glycogen and phosphorylated-protein signals but lower protein- and nucleic-acid–related bands compared with controls—differences that likely reflect sample type and preparation (serum vs. fixed PBS) rather than inconsistency in the underlying biochemical discrimination (Lee et al. [25]).

Clinical applications of FTIR have also extended beyond diagnosis toward monitoring treatment response. FTIR microspectroscopy has been successfully used to follow biochemical shifts during chemotherapy in childhood ALL, showing early decreases in nucleic-acid–associated phosphate bands (e.g., ~1084 cm^−1^) and reductions in carbohydrate content; lipid proxies (cholesterol at ~1467 cm^−1^) showed patient-dependent trajectories (initial decrease in B-ALL, increase then stabilization in T-ALL) (Mordechai et al., 2005 [26]).

At the cellular level, earlier investigations have reported that leukemic leucocytes exhibit elevated nucleic-acid–to-protein indices (e.g., increased PO_2_^−^-associated bands and higher DNA/amide II ratios) compared to normal cells, reinforcing the link between FTIR spectral changes and malignant cell proliferation (Liu, Xu, & Scott, 2006 [27]). Moreover, FTIR analyses of leukemic bone marrow have revealed characteristic alterations in protein secondary structure—most notably a shift from α-helices toward an increased contribution of anti-parallel *β*-sheet relative to controls (Raouf et al., 2009 [28]). Together, these cellular- and tissue-level observations align with our serum-based results, in which amide I/II intensities are altered, suggesting that systemic biofluid spectra can capture underlying cellular-level changes.

Studies using leukemic and lymphoma cell lines have demonstrated that FT-IR spectroscopy, combined with multivariate analysis, can differentiate hematological malignancies based on intrinsic biochemical fingerprints. Babrah et al. reported clear spectral differences across T-cell lymphoma, B-cell lymphoid, and myeloid leukemia lines in the 1800–900 cm^−1^ fingerprint region (proteins: amide I ~1655 cm^−1^, amide II ~1549 cm^−1^; lipids ~1452 cm^−1^; nucleic acids ~1091/1065/972 cm^−1^) and achieved high sensitivities (74–100%) and specificities (≈94–100%) using a PCA-fed LDA classifier with leave-one-out cross-validation [29]. In an earlier report, the same group showed reproducible clustering and ~95% training performance with sensitivities 94–99% and specificities 98–100% across five cell-line classes [29,30]. Consistent with these studies, our data show elevated absorbance near ~1080 cm^−1^ (sym. PO_2_^−^), reinforcing the diagnostic utility of nucleic-acid–related bands and nucleic-acid/protein ratios in distinguishing leukemic from non-leukemic profiles.

Expanding beyond experimental models, FTIR has also been applied to patient-derived material. In a small patient series, significant intensity differences were observed between leukemic (ALL/AML/CLL/CML) and normal blood in the amide I/II region and lipid-associated bands (e.g., ~1454 cm^−1^), with simple lipid and protein intensity ratios (I_2091_/I_3450_ and I_1548_/I_1650_) differing from normal—supporting the utility of protein/lipid balance for discrimination (Alamin H. J. A., 2011 [31], Chandramalar M., 2017 [32])). Likewise, FTIR microspectroscopy of leukemic patient lymphocytes revealed decreases in carbohydrate and phosphate bands and a shift in the ~1082 cm^−1^ PO_2_^−^ band, with post-chemotherapy spectra moving toward normal profiles (Huleihel et al., 2003 [33]). These findings are consistent with the biochemical changes we observe in serum and highlight FTIR’s potential to track therapeutic responses.

Beyond diagnosis, FTIR has also been explored as a method for detecting minimal residual disease (MRD). In a single-patient case report using bone-marrow samples, Raouf and Al Jaouni demonstrated that alterations in amide and phosphate bands—together with shifts in the amide I band and diagnostic intensity ratios—could flag early relapse in pediatric ALL, offering a spectral framework for MRD monitoring (Raouf & Al Jaouni, 2010 [34]).

Across diverse sample types—serum/biofluids, peripheral blood smears, patient cells, bone marrow, and cell lines—FTIR reproducibly captures leukemia-associated biochemical patterns, most notably shifts in amide I/II and nucleic-acid regions alongside changes in the protein–lipid balance. In our pediatric serum cohort we observe the same signature, extending it in a coherent clinical setting and supporting FTIR’s potential as a minimally invasive adjunct for screening. The specific features identified here—altered protein-to-lipid ratios and increased nucleic-acid–related absorbance—map onto core cancer hallmarks (dysregulated metabolism, uncontrolled proliferation, heightened nucleic-acid turnover), strengthening confidence that FTIR reflects disease biology rather than nonspecific noise.

From a clinical-utility perspective, the high sensitivity observed in threshold-adjusted models suggests that, if confirmed in independent cohorts and supported by standardized pre-analytical and analytical procedures, FTIR could serve as a supportive, front-end screening aid performed in parallel with routine tests (complete blood count and basic biochemistry). In practice, it would return a simple risk flag (e.g., “low” vs. “high” ALL probability) to prioritize hematology referral. In this role, FTIR would complement—rather than replace—standard diagnostics and could help identify at-risk children earlier in the diagnostic pathway.

Despite these promising aspects, the present work has important constraints that temper immediate clinical translation. As a single-center study with a modest sample size, it is best viewed as proof-of-principle; its estimates, though statistically robust under cross-validation, require confirmation in independent, externally validated cohorts to establish generalizability. Moreover, our analysis focused on pediatric ALL; pediatric AML—approximately 20% of childhood leukemias—was not included because too few cases were available at our site. Although ALL is the most common childhood cancer, pediatric malignancies overall are rare/ultra-rare, with low annual incidence, which limits recruitment—particularly in single-center studies. Within this context, however, our dataset represents, to the best of our knowledge, the largest pediatric ALL cohort analyzed by FTIR reported in the available literature.

Biologically, serum FTIR captures a composite signature that integrates tumor-derived signals with host responses. Inflammation, nutritional status, and intercurrent infection can modulate the spectral profile and thereby dilute disease specificity. FTIR spectroscopy captures a composite biochemical signature and cannot resolve the relative contributions of specific pathophysiological mechanisms (e.g., inflammation vs. tumor burden, or metabolic reprogramming vs. nutritional factors). Future studies integrating FTIR with targeted proteomics, metabolomics, and clinical biochemistry would help elucidate the molecular origins of the observed spectral changes and strengthen biological interpretation. Disentangling leukemia-specific changes from those shared with other hematologic or metabolic conditions will require carefully designed control groups, standardized sampling windows, and harmonized pre-analytical handling to minimize avoidable variance. The control group in this study included both healthy children and those with benign hematologic abnormalities, allowing evaluation of FTIR performance against clinically relevant differential diagnoses. turnover -associated spectral alterations. Nevertheless, future studies will include additional controls (e.g., infectious or inflammatory conditions) to further confirm disease specificity.

An additional limitation is that we did not explore correlations between FTIR-derived spectral features and clinical parameters such as blast percentage, MRD status, or immunophenotypic subtype (B-ALL vs. T-ALL). While these data were available for the cohort, the modest sample size—particularly for subgroup comparisons (e.g., *n* = 5 T-ALL)—limited statistical power for robust correlation analyses. Future studies with larger, clinically annotated cohorts should investigate whether FTIR signatures scale with disease burden, correlate with MRD kinetics, or differ across immunophenotypic subtypes, as such analyses would strengthen biological interpretation and clarify the potential role of FTIR beyond initial screening.

Methodologically, logistic regression provided an interpretable baseline that performed well in our data; nonetheless, more advanced machine- and deep-learning approaches may further improve discrimination. Their utility, however, hinges on access to larger datasets to mitigate overfitting and on the use of transparent, explainable modeling frameworks to sustain clinical trust. Recent advances in quantitative FTIR-based analysis have also demonstrated the reproducibility and predictive capacity of serum spectroscopy. Chechekina et al. [35] developed regression models for predicting major biochemical parameters of blood serum (including proteins, lipids, and enzymes) using Random Forest Regression, achieving coefficients of determination exceeding R^2^ = 0.95. Their findings confirm the feasibility of reagent-free, quantitative biofluid analysis and highlight the high reproducibility of serum FTIR spectroscopy. This quantitative perspective complements classification-oriented screening studies, such as ours, and collectively strengthens confidence in FTIR as a robust clinical diagnostic platform. In parallel, technical standardization remains critical: drying and storage, instrument calibration, and preprocessing choices (e.g., baseline correction, normalization) materially affect spectral outputs. Addressing water-band interference and adopting shared standard operating procedures SOPs, reference materials, and inter-laboratory comparisons will be essential for reproducibility.

Ultimately, successful translation will depend on demonstrating not only accuracy but also reproducibility, scalability, and cost-effectiveness in real-world care. Prospective, multi-center trials—with head-to-head comparisons against current diagnostic standards and clearly defined decision thresholds and intended-use scenarios—are needed to determine FTIR’s true added value in pediatric leukemia pathways.

## 5. Conclusions

In a single-center pediatric cohort, serum FTIR spectroscopy differentiated ALL from non-leukemic controls with cross-validated AUCs ~0.80 and reproducible, partial separation in unsupervised analyses. Spectral changes—elevated amide I/II and ~1080 cm^−1^ nucleic-acid bands with reduced lipid signatures—are biologically consistent with leukemic metabolic reprogramming. Beyond discrimination, we deliver a standardized, replicable workflow (sample handling, preprocessing, and cross-validation) that proved stable across replicates, underscoring analytical robustness. These data support FTIR as a rapid, minimally invasive, low-cost adjunct to current diagnostics with potential for early triage. External multi-center validation, protocol harmonization, and prospective, real-world evaluation (including integration with routine tests) are needed to establish specificity, scalability, and clinical impact.

## Figures and Tables

**Table 1:** Clinical characteristics of the FT-IR study cohort. Legend: HC = healthy controls; HEMC = non-ALL hematology controls; LEUK-PD = pediatric acute lymphoblastic leukemia. Numbers correspond to patients included in the FT-IR serum analysis (*N* = 103).

Group	No of Patients	Female/Male	Age (Years), Mean [Range]	Diagnoses
LEUK-PD (ALL)	45	22/23	7.3 [1–17.6]	B-ALL (43), T-ALL (5); median blasts 85%
HEMC (hematology controls)	44	20/24	10.5 [1.3–18.0]	anemia (6), thrombocytopenia (13), leukopenia (16), pancytopenia (8), other (1)
HC (healthy controls)	14	7/7	10.5 [0.5–18]	Healthy children
TOTAL	103			

**Table 3:** Classification metrics at selected probability thresholds for discrimination of LEUK-PD vs. CONTROLS (HC + HEMC). Metrics were calculated from out-of-fold predictions obtained in 10-fold stratified cross-validation using balanced logistic regression. Reported values include sensitivity, specificity, precision, accuracy, and F1-score, together with corresponding confusion matrix counts (TP, FP, TN, FN). This illustrates the effect of varying the decision threshold, highlighting the trade-off between sensitivity and specificity relevant for screening applications.

Threshold	Sensitivity	Specificity	Precision	Accuracy	F1	TP	FN	TN	FP
0.50	0.733	0.707	0.660	0.718	0.695	33	12	41	17
0.47	0.778	0.707	0.673	0.738	0.722	35	10	41	17
0.40	0.800	0.638	0.632	0.709	0.706	36	9	37	21
0.30	0.844	0.552	0.594	0.680	0.697	38	7	32	26

**Table 4:** Classification performance of different algorithms for discrimination between LEUK-PD and controls based on preprocessed FTIR spectra. Logistic Regression values correspond to the optimal threshold of 0.47 (as reported in Table 3). All models were evaluated using stratified 10-fold cross-validation. Performance metrics include accuracy, sensitivity, specificity, precision, F1 score, ROC AUC, balanced accuracy, and Matthews correlation coefficient (MCC).

Model	Accuracy	Sensitivity	Specificity	Precision	F1	ROC AUC	Balanced Acc	MCC
Logistic Regression	0.738	0.778	0.707	0.673	0.722	0.800	0.743	0.46
SVM (linear)	0.680	0.622	0.724	0.630	0.629	0.739	0.673	0.35
Random Forest	0.718	0.600	0.810	0.660	0.651	0.805	0.705	0.42
LDA	0.786	0.711	0.845	0.740	0.744	0.804	0.778	0.56

## Data Availability

The data presented in this study are available on request from the corresponding author due to legal reasons.

## Supplementary Materials

The following supporting information can be downloaded at: https://www.mdpi.com/article/10.3390/cancers17213548/s1. Figure S1. Distribution of PC1 scores for LEUK-PD (red) and CONTROLS (blue). Although the mean values did not differ significantly (Welch’s *t*-test p = 0.82), the overall PCA pattern (Figure 5) indicated systematic divergence, suggesting that discrimination arises from a multivariate rather than a univariate signal. Figure S2. PC1 loadings across the FT-IR wavenumber range. The most influential regions correspond to protein-associated amide I (~1640 cm^−1^) and amide II (~1545 cm^−1^) bands, as well as lipid-associated CH_2_ vibrations (~1450 and 2920 cm^−1^), consistent with the biochemical interpretation of supervised results. Figure S3. Heatmap of PC scores (PC1–PC5) across all samples. Rows represent individual samples (grouped by label: red = LEUK-PD, blue = CONTROLS), columns represent principal components. Supplementary Methods and Results – Replicate-level prediction variability: Figure S4–S6. Bland–Altman plots for predicted probabilities (replicate pairs 1–2, 1–3, 2–3). Figure S7–S9. Bland–Altman plots for predicted logits (replicate pairs 1–2, 1–3, 2–3). Figure S10. Boxplot of per-patient RSD (%) of probabilities by class. Figure S11. Boxplot of per-patient SD of logits by class. Supplementary Table S1 (Excel file). Per-patient replicate variability metrics (probability RSD%; logit SD). ICC summary (absolute agreement; ICC (2,1) and ICC (3,1)) with bootstrap 95% CIs. Bland–Altman statistics (bias, SD of differences, 95% LOA) for replicate pairs. Per-replicate out-of-fold predictions (probabilities and logits) for all samples. Distinct score profiles between groups highlight that multiple PCs contribute to the biochemical differentiation of leukemia and controls.

## Abbreviations

ALL	Acute Lymphoblastic Leukemia
ATR	Attenuated Total Reflection
AUC	Area Under the Curve
AsLS	Asymmetric Least Squares
BM	Bone Marrow
CaF_2_	Calcium Fluoride
EDTA	Ethylenediaminetetraacetic Acid
FDR	False Discovery Rate
FTIR/FT-IR	Fourier-Transform Infrared (Spectroscopy)
HC	Healthy Controls
HEMC	Hematology Controls (non-ALL)
HCA	Hierarchical Cluster Analysis
IC-BFM	International Berlin-Frankfurt-Münster protocol
LDA	Linear Discriminant Analysis
LEUK-PD	Pediatric Leukemia (ALL patients at diagnosis)
MCC	Matthews Correlation Coefficient
MRD	Minimal Residual Disease
MCT	Mercury Cadmium Telluride (detector)
OPUS	Operating Program for Universal Spectroscopy (Bruker software)
OOF	Out-Of-Fold (predictions)
PCA	Principal Component Analysis
PBS	Peripheral Blood Smear
PR	Precision–Recall
ROC	Receiver Operating Characteristic
SAA	Severe Aplastic Anemia
S-Monovette	Safety-Monovette (blood collection system, Sarstedt)
SNV	Standard Normal Variate (normalization)
SVM	Support Vector Machine
