# Supplementary material for: Serum Fourier-Transform Infrared Spectroscopy with Machine Learning for Screening of Pediatric Acute Lymphoblastic Leukemia: A Proof-of-Concept Study
Source: Cancers (Basel). 2025 Nov 1;17(21):3548. doi: 10.3390/cancers17213548 (PMC12606736; doi:10.3390/cancers17213548)
Supplement: Supplementary file 1 [file cancers-17-03548-s001.zip › Supplementary Table S1.pdf]

BAND (CM^{-1})	ASSIGNMENT	MAX Δ (AU)	% VARIATION
**1080**	Glycogen	0.0031	0.31%
**1450**	CH_2 deformation	0.0059	0.59%
**1545**	Amide II	0.0042	0.42%
**1640**	Amide I	0.0028	0.28%
**2920**	Lipids	0.0067	0.67%

Table S1. *Baseline correction stability validation. Quantitative comparison between two-stage (rubberband+AsLS) and single-step AsLS baseline corrections at key biomarker regions. Max Δ = maximum absolute difference; AU = absorbance units.*
