# Supplementary figures and images for: Serum Fourier-Transform Infrared Spectroscopy with Machine Learning for Screening of Pediatric Acute Lymphoblastic Leukemia: A Proof-of-Concept Study
Source: Cancers (Basel). 2025 Nov 1;17(21):3548. doi: 10.3390/cancers17213548 (PMC12606736; doi:10.3390/cancers17213548)

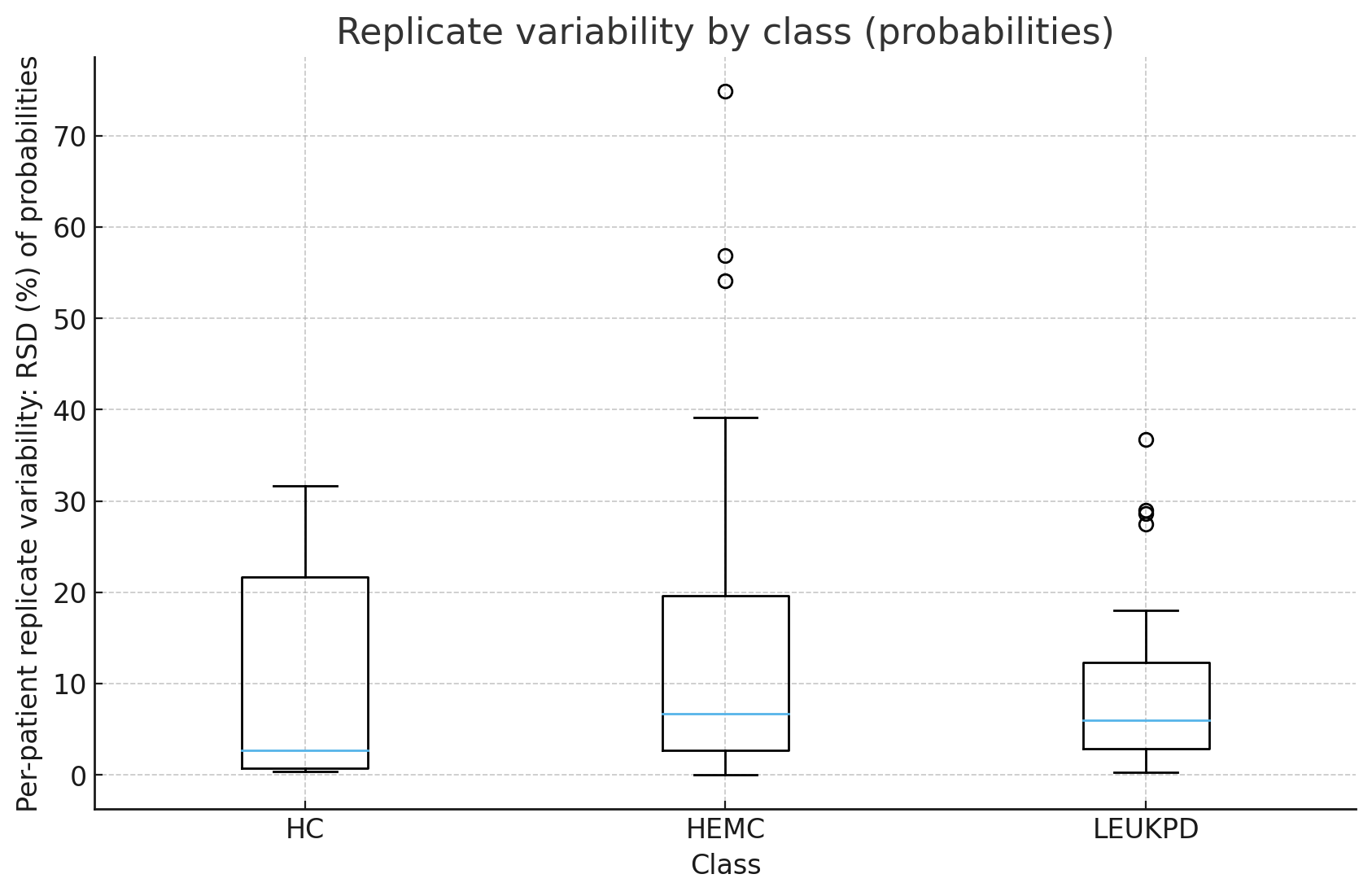

Supplement: Supplementary file 1 [file cancers-17-03548-s001.zip › Fig. S10. Boxplot_probability_RSD_by_class_EN.png]

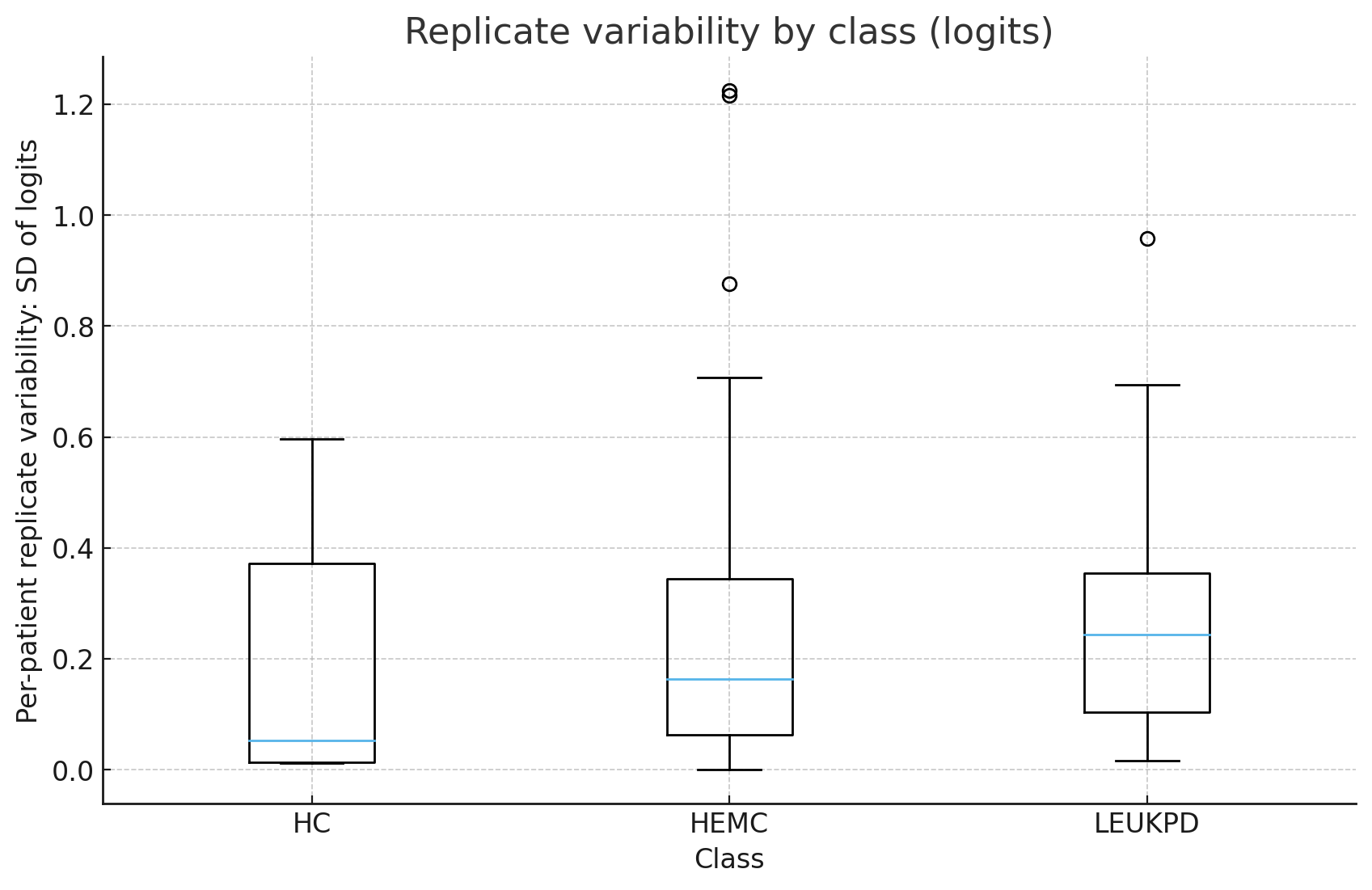

Supplement: Supplementary file 1 [file cancers-17-03548-s001.zip › Fig. S11. Boxplot_logit_SD_by_class_EN.png]

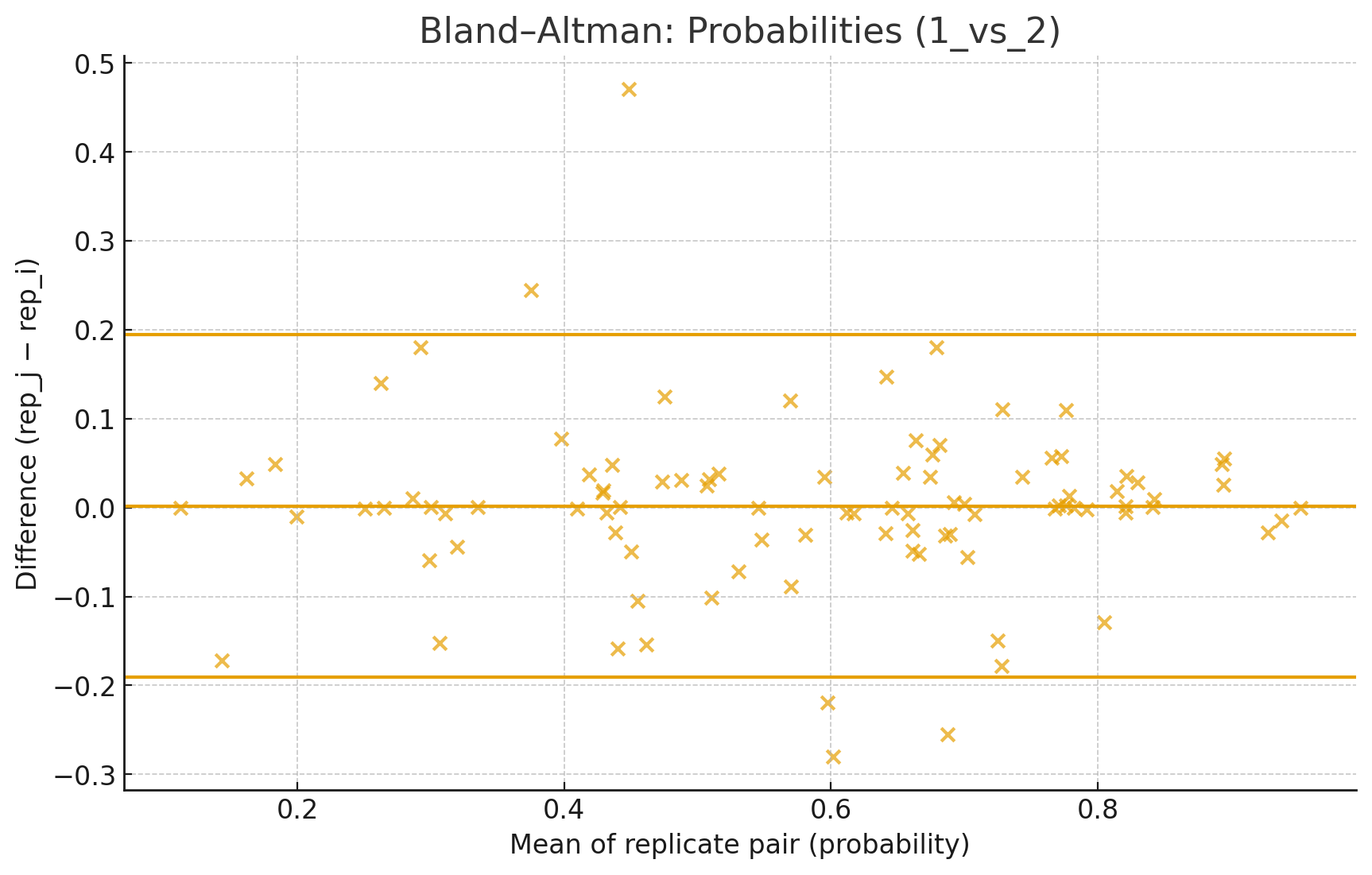

Supplement: Supplementary file 1 [file cancers-17-03548-s001.zip › Fig. S4. BA_probability_1_vs_2_EN.png]

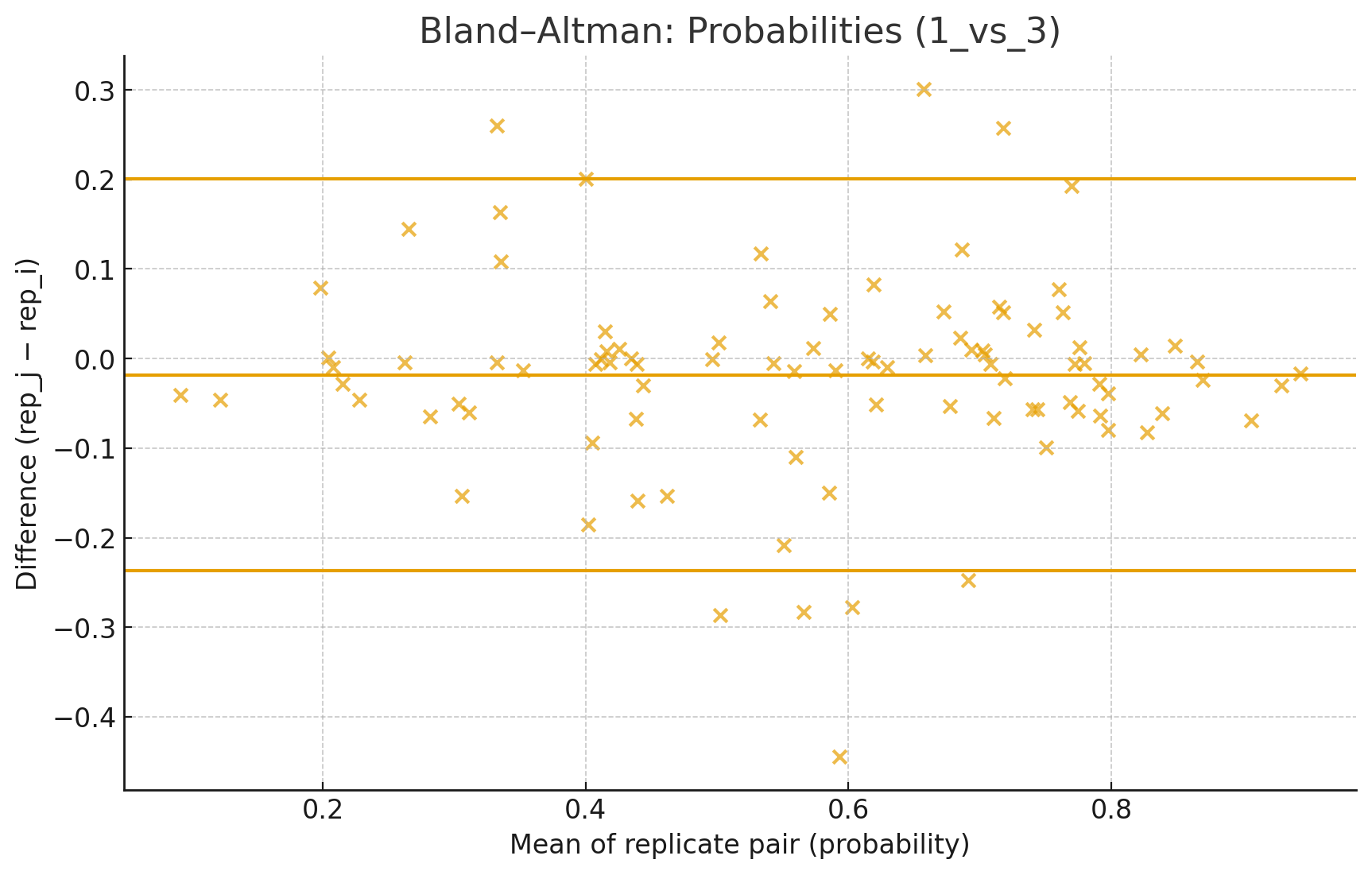

Supplement: Supplementary file 1 [file cancers-17-03548-s001.zip › Fig. S5. BA_probability_1_vs_3_EN.png]

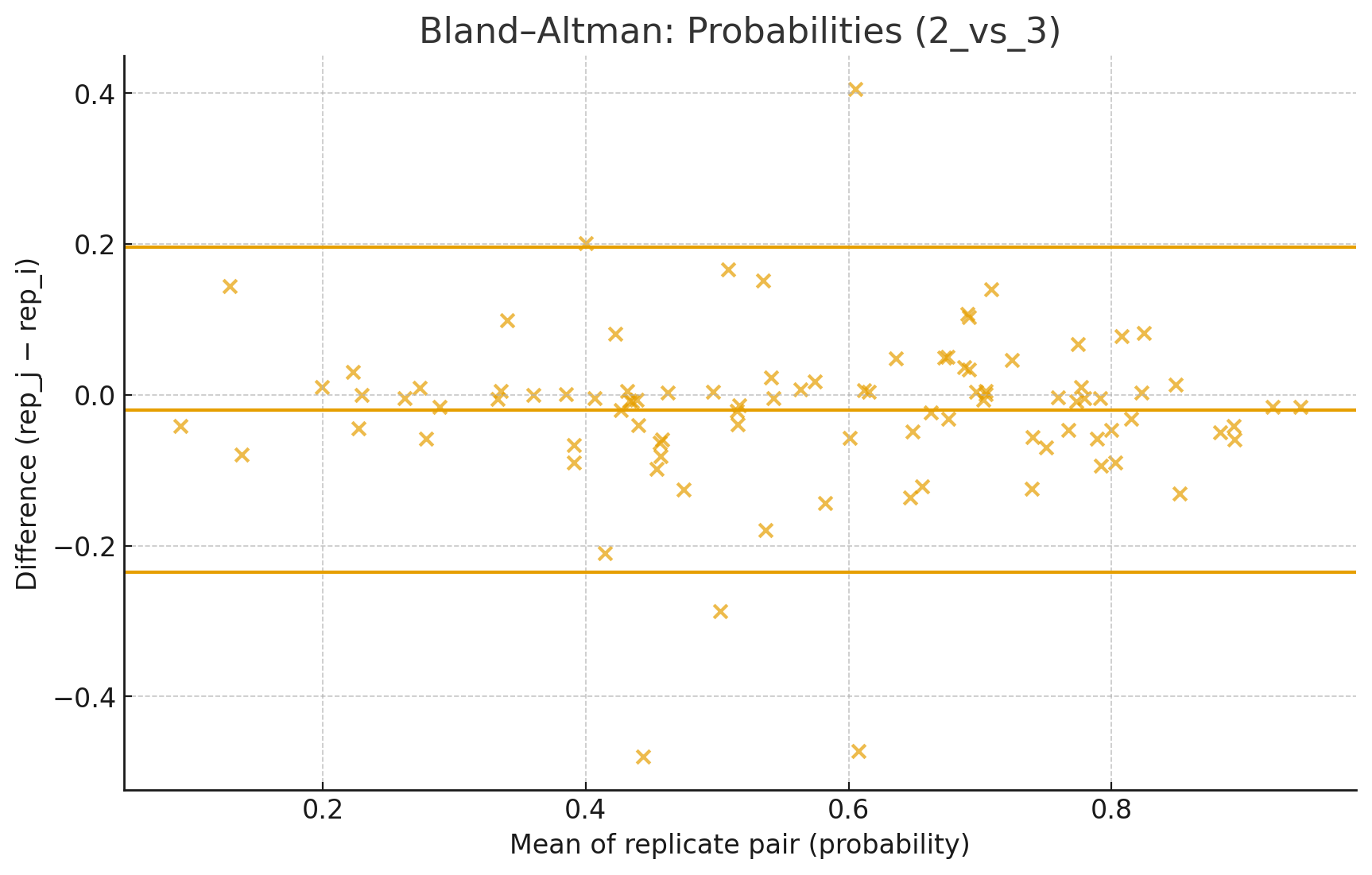

Supplement: Supplementary file 1 [file cancers-17-03548-s001.zip › Fig. S6. BA_probability_2_vs_3_EN.png]

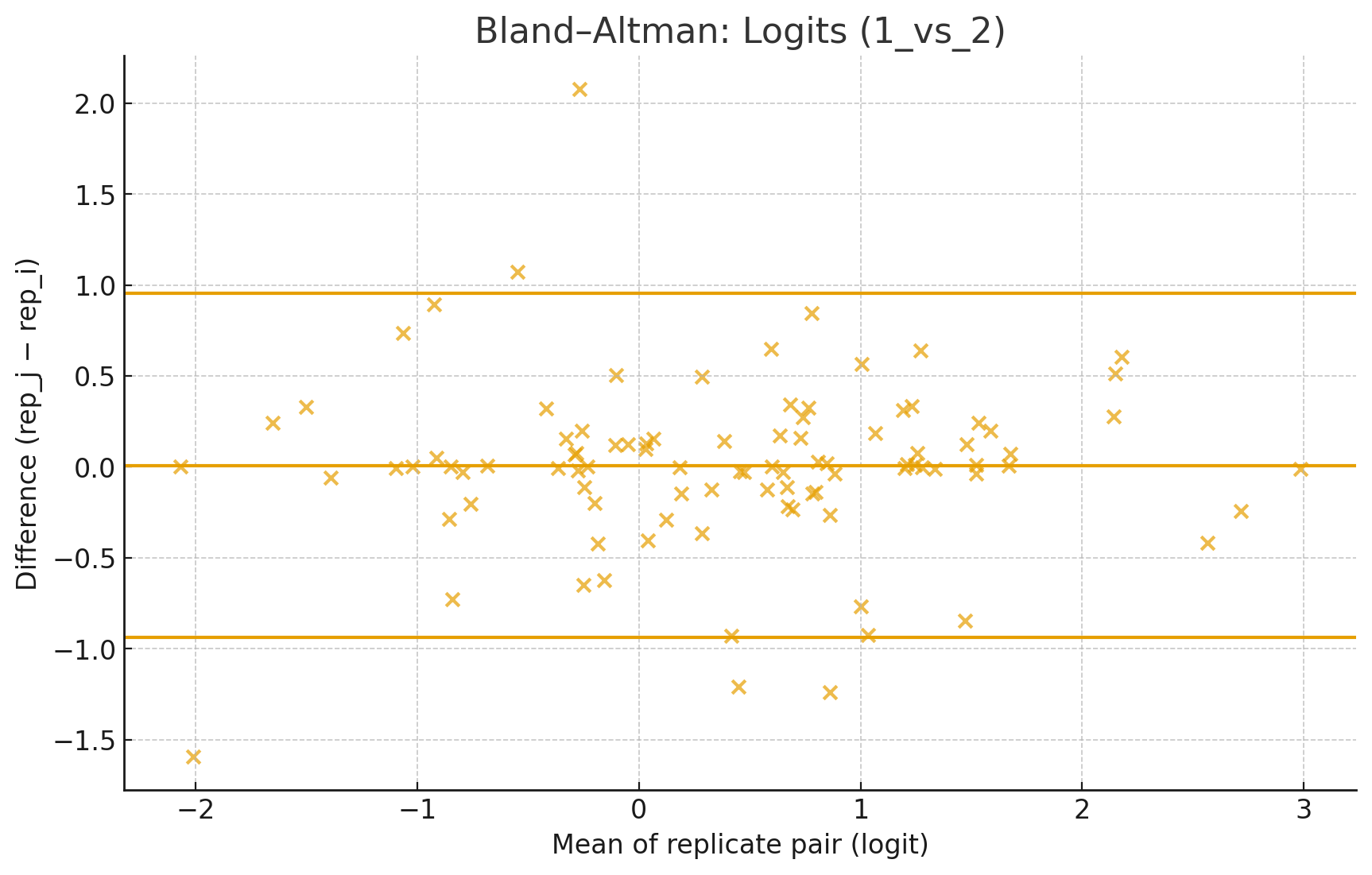

Supplement: Supplementary file 1 [file cancers-17-03548-s001.zip › Fig. S7. BA_logit_1_vs_2_EN.png]

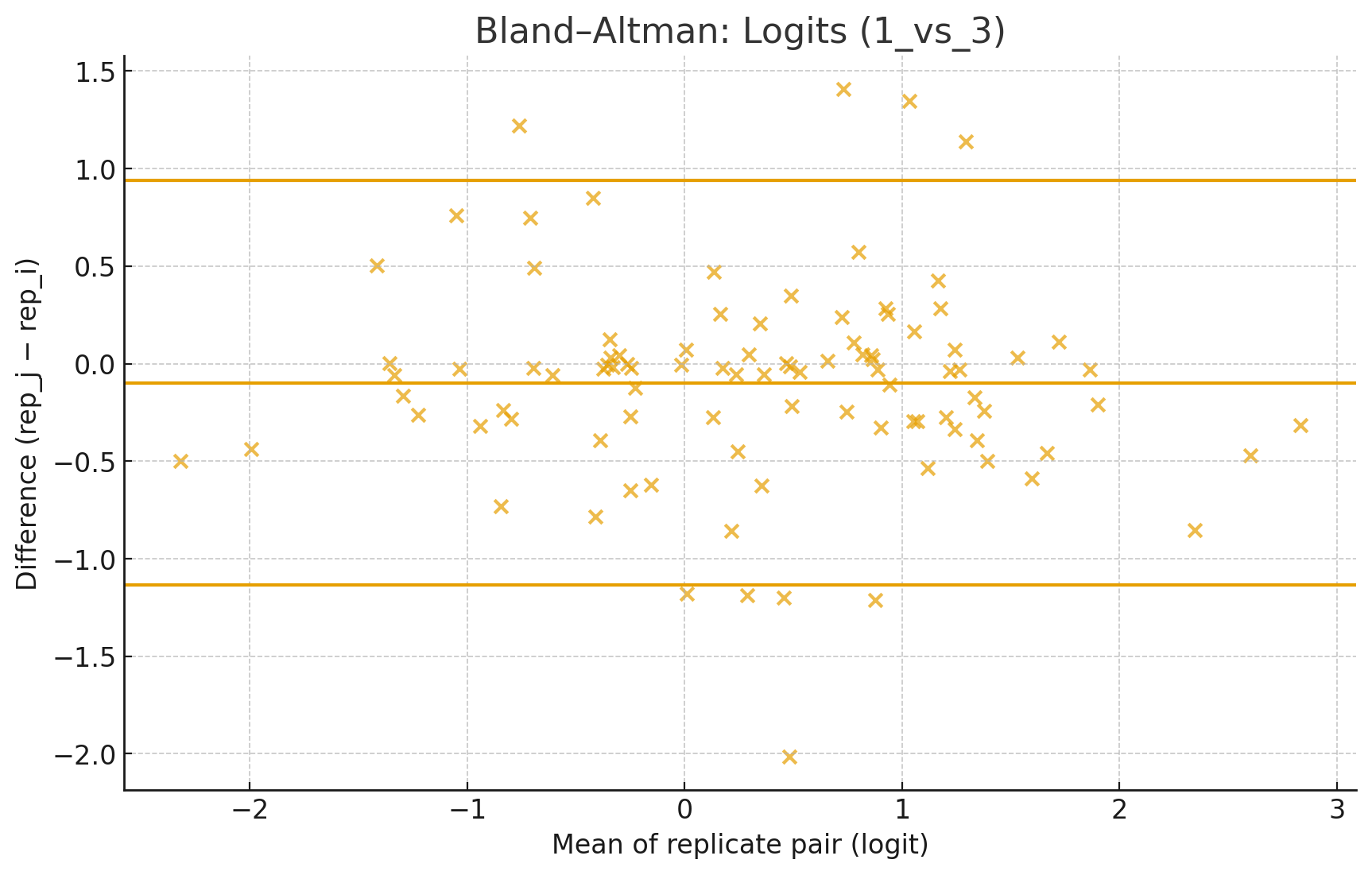

Supplement: Supplementary file 1 [file cancers-17-03548-s001.zip › Fig. S8. BA_logit_1_vs_3_EN.png]

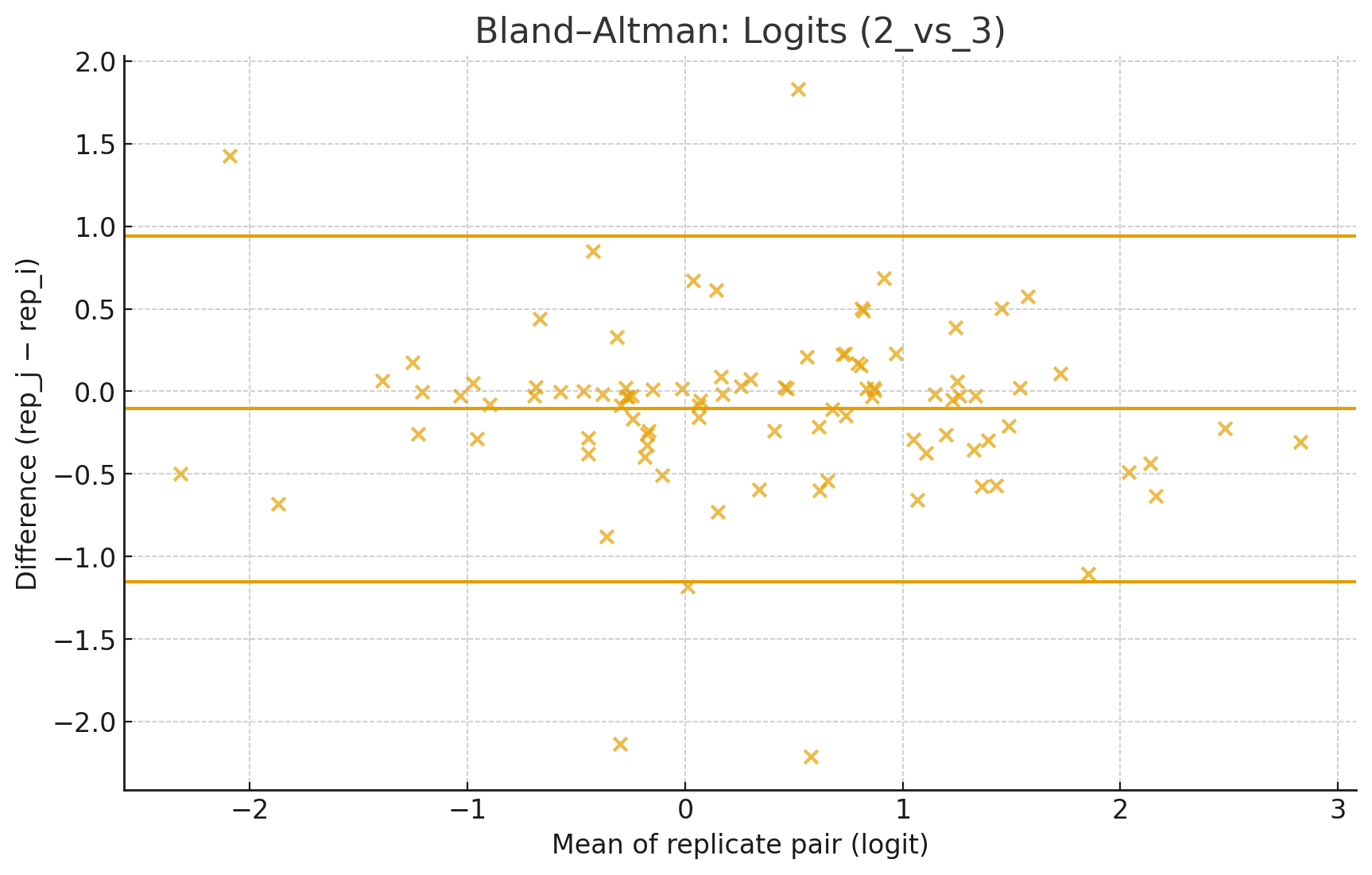

Supplement: Supplementary file 1 [file cancers-17-03548-s001.zip › Fig. S9. BA_logit_2_vs_3_EN.png]

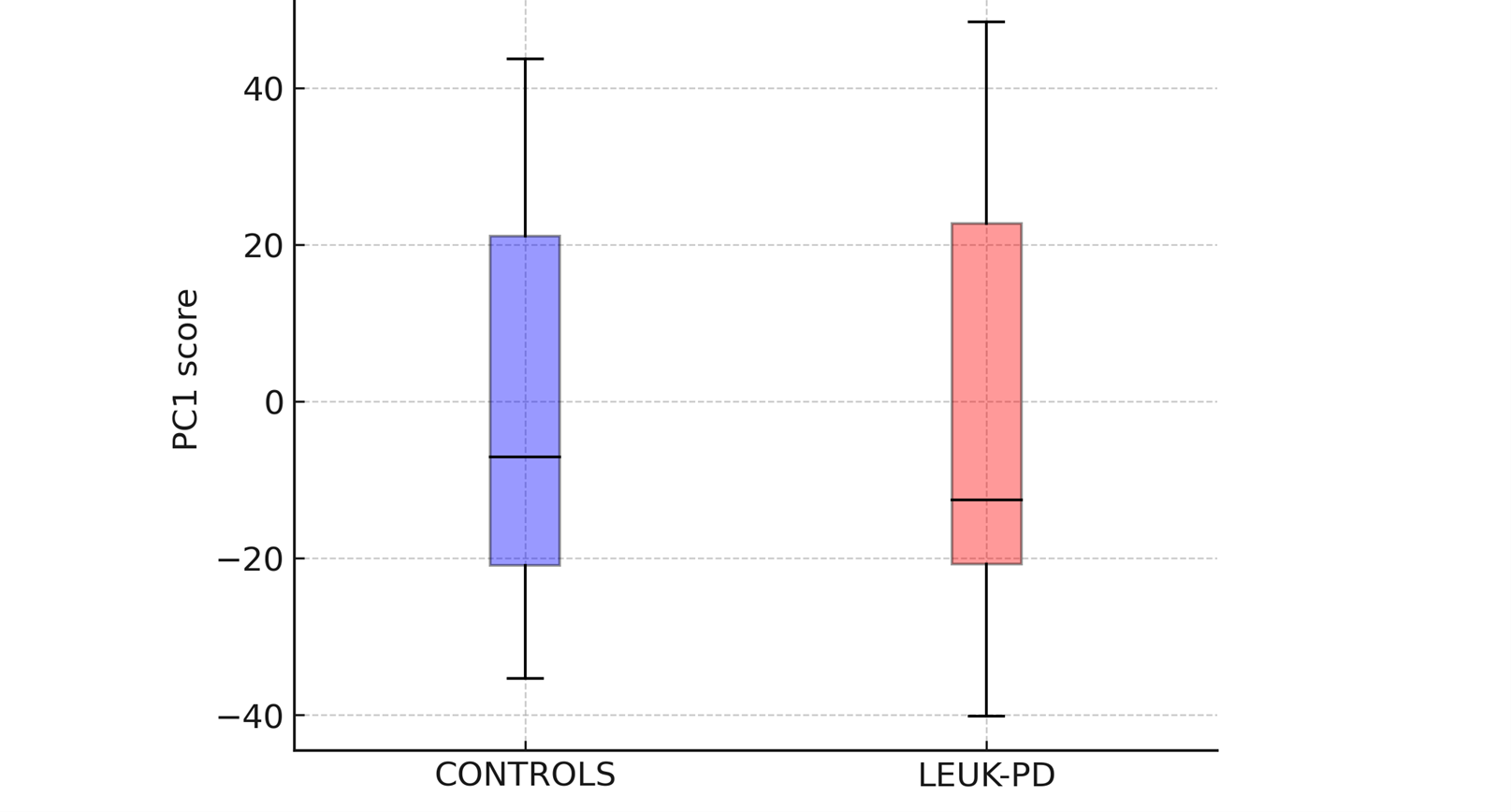

Supplement: Supplementary file 1 [file cancers-17-03548-s001.zip › Supplementary Figure S1.png]

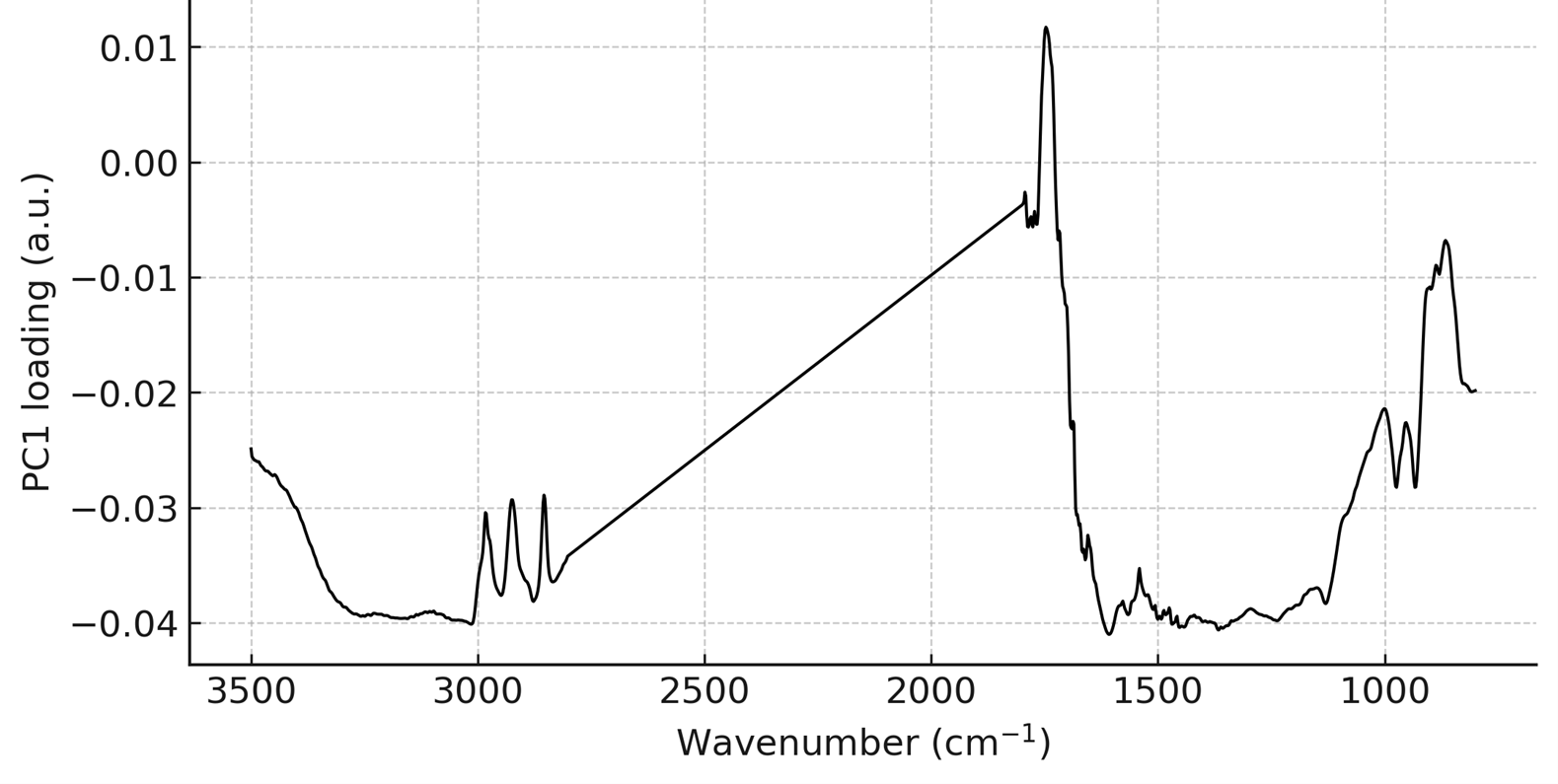

Supplement: Supplementary file 1 [file cancers-17-03548-s001.zip › Supplementary Figure S2.png]

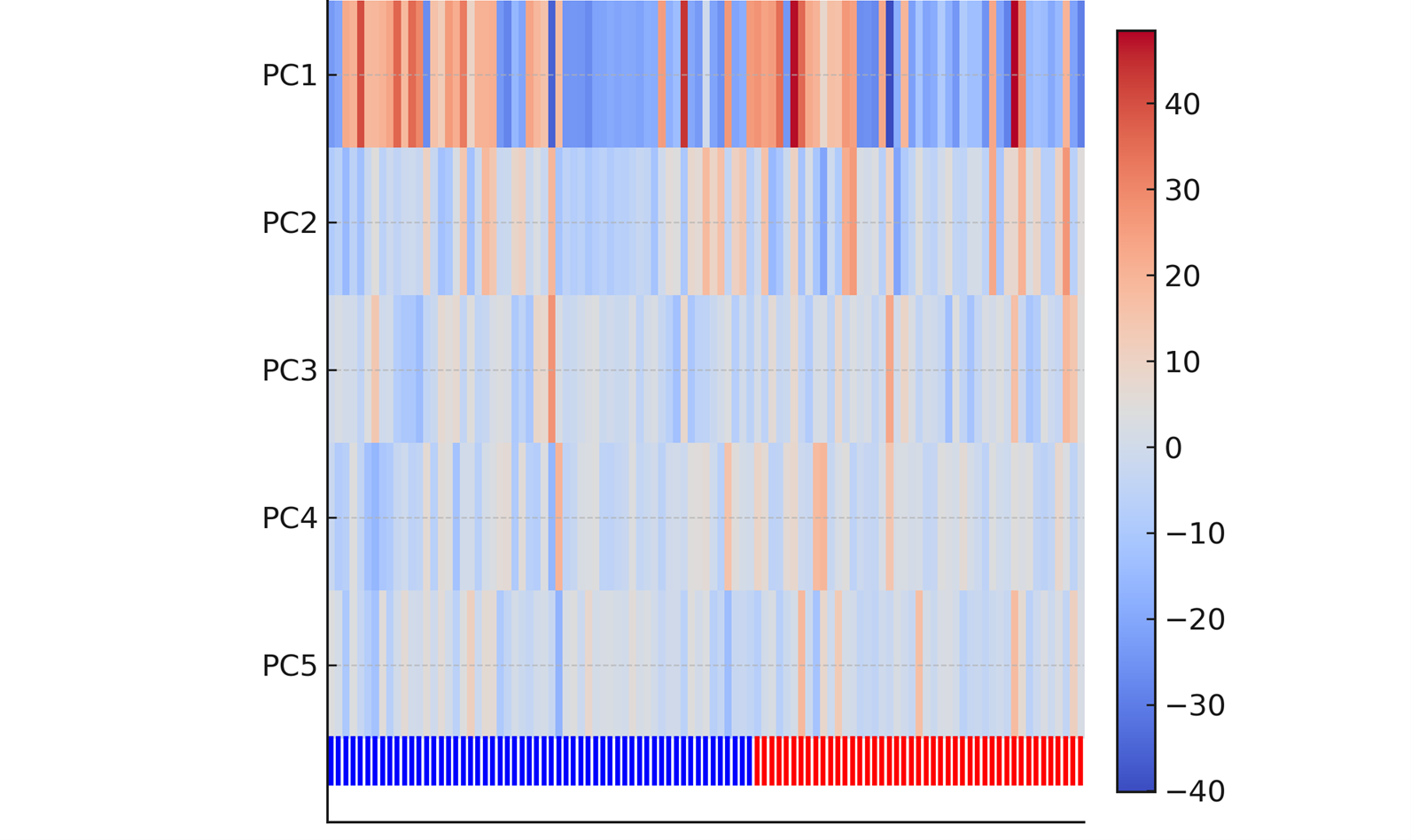

Supplement: Supplementary file 1 [file cancers-17-03548-s001.zip › Supplementary Figure S3.png]
